# Religion and Emotion Regulation: A Systematic Review of Quantitative Studies

**DOI:** 10.1007/s10943-024-02216-z

**Published:** 2025-02-06

**Authors:** Tânia Brandão

**Affiliations:** William James Center for Research, Ispa – Instituto Universitário, R. Do Jardim Do Tabaco 34, Lisbon, Portugal

**Keywords:** Religion, Spirituality, Emotion regulation, Systematic review

## Abstract

The association between religion/spirituality and emotion regulation has been the subject of growing interest in the last years since studies have suggested that emotion regulation is likely to be shaped by religion/spirituality. The purpose of this systematic review was to synthesize the quantitative empirical studies toward understanding the relationship between religion/spirituality and emotion regulation. Database searches were conducted in different databases from inception to March 2022 using relevant search terms. Quantitative studies exploring the role of religion/spirituality on emotion regulation were included in this review. Of 887 abstracts identified only 15 studies were deemed eligible for inclusion. Studies were organized in terms of associations between religion/spirituality and emotion regulation according to religious affiliation, associations between religion/spirituality and emotion regulation regardless of religious affiliation, and emotion regulation as a mediator between religion/spirituality and several psychological-related outcomes. Overall, the findings revealed small to moderate associations between religion and emotion regulation, with correlation coefficients ranging from 0.13 to 0.50 for cognitive reappraisal, 0.08 to − 0.72 for expressive suppression, and − 0.09 to 0.56 for other emotion regulation dimensions. Furthermore, most studies highlighted emotion regulation as a key mechanism linking religion/spirituality to psychological outcomes across diverse contexts. Differences in emotion regulation strategies have been examined in a few religious affiliations and most of the studies explored the link between religion/spirituality and emotion regulation regardless of religious affiliation.

## Introduction

*Religion* refers to “institutionalized or culture-bound ways of relating to that which is perceived to be sacred” (Rosmarin & Koenig, 2020, p. xix), and includes beliefs, practices, and rituals related to the divine (Koenig, 2018). More recently, it has been integrated as a dimension of *spirituality*, a broader construct that refers “to any way of relating to that which is perceived to be sacred” (Rosmarin & Koenig, 2020, p. xix). Both dimensions are interrelated since many individuals believe in a sacred figure, but do not report be affiliated with a religious group (Rosmarin & Koenig, 2020). Both religion and spirituality integrate two dimensions: observational behaviors (e.g., prayer, participation in rituals or religious service) and cognitive dimensions (e.g., faith) (Rosmarin & Leidl, 2020) and are likely to influence individuals’ thoughts, feelings, and behaviors (Vishkin et al., 2016).

Specifically, religion has been related to great emotional experiences (Emmons, 2005). It is now recognized that religion can influence and shape not only emotion generation but also emotion goals and emotion regulation, and especially the experience and expression of emotions (Emmons, 2005; Vishkin et al., 2014). *Emotion regulation* (ER) refers “to the processes by which individuals influence which emotions they have when they have them, and how they experience and express these emotions” (Gross, 1998, p. 275). ER has been assessed in different ways: in terms of (cognitive) ER strategies (e.g., cognitive reappraisal, rumination), overall ER (e.g., expression of emotions, empathy, and emotional self-awareness), and overall difficulties in ER (e.g., difficulties in emotional awareness or acceptance) (Garnefski & Kraaij, 2007; Gratz & Roemer, 2004; Gross & John, 2003; Shields & Cicchetti, 1995).

It is hypothesized that religion/spirituality influences emotions through different pathways (Silberman, 2003). One would be through the prescription of emotions in terms of their appropriateness and intensity; the other would be through beliefs about the sacred that contribute to experiencing specific emotions capable of impacting individuals’ well-being in general; and the last one would be through offering an opportunity to experience emotions related to the sacred (Silberman, 2003). Other authors pointed out the influence of religion/spirituality on emotions’ intensity by influencing the event-related meaning, deservedness, and controllability (Ben-Ze’ev, 2002). As suggested by several authors, religion can set the stage for ER by teaching how to handle intense and unpleasant emotions (Schimmel, 1997; Watts, 1996).

Recently, Vishkin (2021) highlighted that religion-specific characteristics and interactions with national context would be responsible for variations and consistencies in beliefs about the controllability of emotions, desired emotions, ER strategies, and intrinsic and extrinsic ER. Thus, it seems that individuals have some patterns of ER that are tied to a particular religion (Vishkin, 2021). For instance, Buddhists in comparison to Protestants tend to use more ER strategies that do not interfere with emotions such as acceptance (Wilken & Miyamoto, 2020). Also, expressive suppression (i.e., individuals’ efforts to inhibit outward emotional expressions) is more common among atheists than in religiously affiliated individuals (Burris, 2022).

Thus, the aim of this study was systematically reviewed and synthesized the quantitative studies toward understanding the relationship between religion/spirituality and ER.

## Method

This systematic review followed the Preferred Reporting Items for Systematic Reviews and Meta-Analyses (PRISMA; Page et al., 2021).

### Eligibility Criteria

We included quantitative research articles published in English, Portuguese, Spanish or French examining the association between religiosity and ER. Exclusion criteria were: (1) validation or psychometric studies; (2) intervention studies; (3) reviews, books, unpublished articles and doctoral theses, commentaries, abstracts of conferences and congresses, and case reports. Studies were also excluded if the authors were not able to retrieve the full text.

### Search Strategy

Online databases were searched (these included: APA PsycInfo, Academic Search Complete, MEDLINE, CINAHL Plus with Full Text, Psychology and Behavioral Sciences Collection, and APA PsycArticles) using EBSCO, from inception to March 2022. Additional manual searching in Google Scholar and references of retrieved studies were also conducted.

The key terms used were the following: religion or religious or religiousness or religions or religiosity or spiritual or spirituality or faith AND emotion regulation or emotion dysregulation or regulation of emotion or emotional regulation.

### Data Extraction

The following data were extracted and coded: year, country, aims, sample, religiosity measure (and dimensions), ER regulation measure (and dimensions), and main findings (see Table 1).

**Table 1:** Studies Characteristics (*N* = 15)

Authors, year, country	Study objectives	Sample characteristics	Design and statistics	Measure to evaluate religiosity	Measure to evaluate ER	Main findings
Aliche et al. (2020) (Nigeria)	Links between religious commitment, ER and social support	210 inpatients (56% women); M age = 34.05. (SD = 10.36)	Cross-sectional—regression	The Religious Commitment Inventory	Emotion Regulation Questionnaire	Interpersonal commitment linked to more CR (r = 0.50, *p* < 0.001), less ES (r = −0.72, *p* < 0.01); intrapersonal commitment showed opposite trends (r = −0.40, *p* < 0.01 for CR and r = 0.43, *p* < 0.001 for ES)
Dolcos et al. (2021) (USA)	Links between religious coping, ER, resilience and distress	203 young adults (155 women); M age = 21.98 (SD = 4.75)	Cross-sectional—mediation	Brief COPE	Emotion Regulation Questionnaire	Religious coping linked to more CP (r = 0.25, *p* < 0.01), less ES (r = −0.14, *p* < 0.05); CR mediated effects on depression/anxiety
Semplonius et al. (2015) (Canada)	Links between involvement in religious activities, ER and social ties	1.132 students (70.5% female) Mage = 19.06 (SD = 0.93)	Longitudinal study	Demographic questions and The Spiritual Transcendence Index	Difficulties in Emotion Regulation Scale	Higher religious activity predicted less ER difficulty (β = −0.06, *p* < 0.05) consequently led to more social ties over time
Wilken (2020) (USA)	ER strategies in Buddhist vs. Protestant	88 Protestants; Mage = 21.31, SD = 4.59) and 59 Buddhists Mage = 23.69, SD = 7.48)	Cross-sectional study	Religious teachings on ER	Teaching on ER	Buddhists (M = 0.84, SE = 0.07) used non-influence strategies more often than Protestants (M = 0.46, SE = 0.10). F(1, 143) = 9.75, *p* = 0.002, partial η2 = 0.06. These were linked to fewer depressive symptoms in both samples
Vishkin (2019a) (Israel)	Links between religiosity, ER, affect, and life satisfaction	288 Jewish Israelis (51% female, Mage = 29.63) (Study 1); 277 Christians Americans (48% female, Mage = 34.74) (Study 2)	Cross-sectional—Mediation	Religiosity scale	Emotion Regulation Questionnaire	Religiosity linked to CR (both groups; r = 0.23, *p* < 0.05; r = 0.13, *p* < 0.05) and to ES (only Christians; r = −.19, *p* < .05); CR mediated outcomes in Christians but not Jews
Holmes (2019) (Greece)	Links between religiousness, ER, and risk-taking	167 adolescents (52% male); M = 14.13, SD = 0.54; 67.5% religion: Protestant 67.5%; Catholic 5%; Jewish 2.5%	Longitudinal—GLM	Religiousness scale	Emotion Regulation Checklist	Religiousness T1 correlated with better ER T2 (r = .22, *p* < .01); ER mediated the link between religiousness and lower risk-taking
Vishkin (2019b) (Israel)	Links between religiosity and ER beliefs and motivations	United States, N = 210 (58.6% female, Mage = 40.64, SD = 11.99); Israel, N = 203 (52.7% female, Mage = 41.62, SD = 12.66); and Turkey, N = 203 participants (36.5% female, Mage = 34.45, SD = 10.56)	Cross-sectional—regression	Religious Commitment Inventory	Several measures	Religiosity linked to more CR (r = .13, *p* < .01), acceptance (r = .11, *p* < .01), less rumination (r = −0.09, *p* < 0.05), more ES (r = 0.08, *p* < 0.05), distraction (r = 0.17, *p* < 0.01). Links did not vary by sample or by dimension of religiosity (except for suppression)
Vishkin (2016) (Israel)	Links between religiosity and ER	Muslim sample from Turkey (N = 270, 77% female, Mage = 20.97). Christian sample from America (N = 277, 48% female, Mage = 34.74) Jewish sample from Israel (N = 288, 51% female, Mage = 29.63)	Cross-sectional and experimental studies—Regression	Religious Commitment Inventory	Emotion Regulation Questionnaire	Religiosity positively linked to CR (r = 0.17, *p* < 0.01;r = 0.15, *p* < 0.05; r = 0.22, *p* < 0.01); Religiosity was not linked to ES in the Muslim and Jewish samples; but was negatively linked to ES in the Christian sample (r = −0.19, *p* < 0.01)
Yadav (2018) (India)	Links between spirituality/religiousness, ER and cyber bullying	490 university students – 61% male, aged between 18 and 25 years	Cross-sectional—mediation	Spirituality/Religiousness was measured with Spiritual well-being scale	Emotional Intelligence scale	Religiosity linked to better self-ER (range r = 0.30, *p* < 0.01 to r = 0.56, *p* < 0.01); ER mediated bullying outcomes
Lee (2021) (USA)	Links between religiosity, ER and gried	100 bereaved American college students (73 women). Mean age of 19.62 years (SD = 1.75). predominately Christian faith (n = 87)	Cross-sectional—moderation	The General Religiousness measure The Spiritual Transcendence Index The Brief COPE	Emotion Dysregulation. The Feeling Card	Religious students reported better emotional calm; Religiousness and spirituality correlated with emotion reactivity (r = −0.30, *p* < .01; r = −0.24, *p* < 0.05) but not with emotion recovery
Burris (2020) (Canada)	Link between atheism and suppression	1059 university students (722 women). 50% Christian, 30% agnostic or nonreligious, and 15% self-identified as atheist	Cross-sectional—ANOVAs	Self-reported religion	Emotion Regulation Questionnaire	Atheists (M = 4.05; SD = 1.25) more likely to use ES than religious individuals (M = 3.66; SD = 1.23) F (2, 1056) 8.11, p .001. No difference in CR
Park (2016) (South Korea)	Links between intrinsic religiosity, ER, and meaning	326 participants; 45% men; M age = 22.13 years (SD = 2.08). Most of the participants were Protestants (62.9%), followed by Catholics (24.5%)	Cross-sectional—SEM	Gorsuch and McPherson’s Revised Intrinsic and Extrinsic Religiosity Scale	Event Related Rumination Inventory. Difficulties in Emotional Regulation Scale	Intrinsic religiosity linked to deliberative rumination (r = 0.18,p < 0.01) but not ER; indirect effects on meaning via deliberate rumination
Singh (2014) (India)	Links between religiosity, ER and well-being	150 students (78 male); M age = 22.91; SD = 1.47)	Cross-sectional—Differential analyses	Universal Religious Orientation Scale	Cognitive Emotion Regulation Questionnaire	Highly religious individuals reported less ER dysfunctionality (M = 27.56) and higher functional ER (M = 31.01) than medium (M = 31.84/26.19) and low religious (M = 31.12/27.37). Post-hoc only between medium and low on both
Akbari (2018) (Iran)	Liks between spiritual health, ER, and quality of life, psychological health, and burnout	18 female and 113 male participants	Cross-sectional—Mediation	Spiritual Well-Being Scale	Emotion regulation—Difficulties in Emotion Regulation Scale	Spiritual health linked to lowe emotion dysregulation (r = 0.52, *p* < 001). ER partially mediated the link with quality of life; and fully mediated link to mental health and burnout
Holmes (2016) (USA)	Links between religiousness, ER and health behaviors	220 adolescents (121 males) Mage = 15.10, SD = 1.57). 70% Protestant, 11% Roman Catholic, 1% Jewish, 5% “none,” and 13% “other.”	Cross-sectional—SEM	Religious Behavior Scale adapted from Christian Religious Internalization Scale	Emotion Regulation Checklist	Higher identification predicted higher self-regulation (b = 0.09, *p* < 0.01) and higher introjection predicted lower self-regulation (b = −0.11, *p* < 0.001); ER mediated effect on health-risk behavior

Statistical symbols: M = mean; SD = standard deviation. Other: ER = emotion regulation

## Results

### Study Selection

The search retrieved 887 records: 470 from APA PsycInfo, 286 from Academic Search Complete, 136 from MEDLINE, 91 from CINAHL Plus with Full Text, 57 from Psychology and Behavioral Sciences Collection, and 44 from APA PsycArticles. Of the 887 results, 305 were duplicates and thus were automatically removed. Title and abstracts of the remaining studies (n = 582) were screened according to the inclusion and exclusion criteria.

**Fig. 1:**
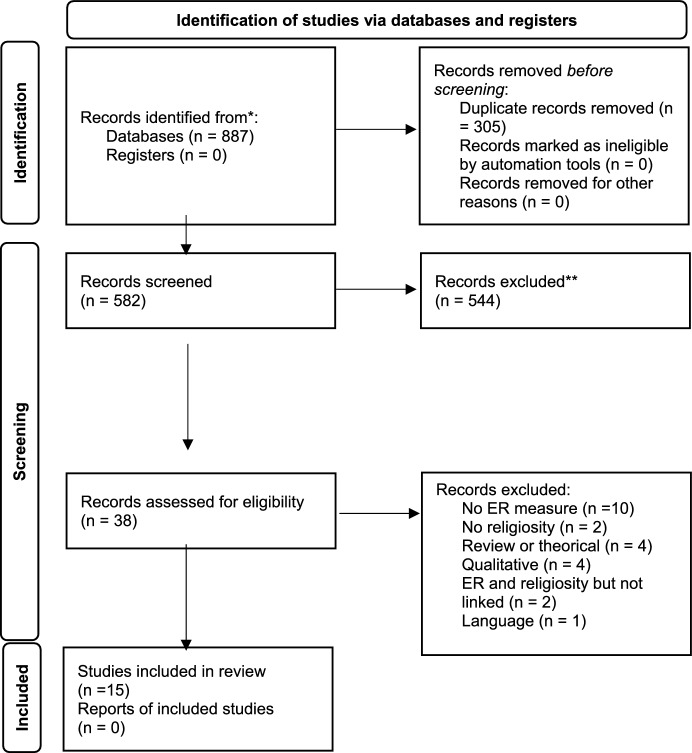
Flow-chart of included studies. *Adapted from:* Page MJ, McKenzie JE, Bossuyt PM, Boutron I, Hoffmann TC, Mulrow CD, et al. The PRISMA 2020 statement: an updated guideline for reporting systematic reviews. BMJ 2021;372:n71. 10.1136/bmj.n71

This screening resulted in 38 studies identified as relevant for full-text reading and 544 identified as irrelevant and for that reason were excluded. From the 38 relevant studies, 23 were excluded (reasons: ten studies did not measure ER; four studies did not measure religiosity; four studies were qualitative; four studies were reviews or theoretical papers; two studies measured ER and religiosity but did not explore the links among them; and 1 study was in a language other than those included in this review). Thus, a total of 15 studies were included in this review. The flow chart with the study selection procedure is displayed in Fig. 1.

### Study Characteristics

Studies were published between 2014 and 2021. Most of the studies were conducted in USA and Israel (n = 4 and 3, respectively), but there was a wide variety of other countries (Canada and India (n = 2), Nigeria, Greece, South Korea, and Iran (n = 1)).

They were mainly cross-sectional in nature (n = 13) and only two were longitudinal. Sample sizes ranged from 100 to 1132. A total of 6148 participants were included in these studies with a mean age of 26.23 years (SD = 8.45) (lowest mean 14.13; highest mean 41.62).

In terms of religiosity assessment, the main measure used was the Religious Commitment Inventory (n = 3) followed by the Spiritual Transcendence Index and the Spiritual Well-Being Scale (n = 2). Four studies developed their own questions regarding religiosity and the following scales were used only by one study (Religious Intending Scale; Brief Cope—religious dimension; Universal Religious Orientation Scale; Intrinsic and Extrinsic Religiosity Scale; The Religiousness Measure; The Brief-Religious Coping Scale; and an adapted version of Christian Religious Internalization Scale).

In terms of ER, the main measure used was the Emotion Regulation Questionnaire (n = 5), followed by the Difficulties in Emotion Regulation Scale (n = 3) and the Emotion Regulation Checklist (n = 2). Other measures used were: Brief Cope (acceptance and reappraisal), the Rumination Reflection Questionnaire, The Thought Control Questionnaire (distraction), the Acceptance and Action Questionnaire (experiential Avoidance), Cognitive-Behavioral Avoidance Scale (behavioral avoidance), Emotional Intelligence Scale, The Feeling Card (emotion dysregulation), The Event-Related Rumination Inventory, and Cognitive Emotion Regulation Questionnaire. The characteristics of the 15 included studies are presented in Supplementary material available online.

### Main Findings: Associations Between Religiosity and ER

Results will be organized in three subsections: (1) associations between religion/spirituality and ER according to religious affiliation and (2) associations between religion/spirituality and ER, regardless of religious affiliation; and (3) and ER as a mediator (linking religion/spirituality to several psychological-related outcomes). Note that, most of the studies included in section three are the same included in section two since first the links between religiosity and ER were measured and then a mediational hypothesis was tested and for that reason, results are presented separately.

#### Associations Between Religion/Spirituality and ER According to the Religious Affiliation

Only five studies explored differences in terms of ER according to religious affiliation (Burris, 2022; Vishkin et al., 2016, 2019a, 2019b; Wilken & Miyamoto, 2020). Specifically, studies showed that Buddhists reported that their religion teaches them to use non-influence strategies of ER and that they use more non-influence strategies of ER than Protestants (Wilken & Miyamoto, 2020).

Also, it was found that religiosity was not associated with expressive suppression (i.e., individuals’ efforts to inhibit outward emotional expressions while still experiencing the underlying emotion internally) in Muslim participants but was significantly associated within Christian and Jewish participants. However, for the Christian sample religiosity was negatively linked to expressive suppression and for the Jewish sample was positively linked to expressive suppression (Vishkin et al., 2016).

In another study, religiosity was positively associated with cognitive reappraisal for both Jewish and Christian samples; however, in terms of expressive suppression, a negative correlation was found only in the Christian sample (Vishkin et al., 2019a, 2019b). A similar pattern was found in another study (Vishkin et al., 2019a, 2019b). In this study, the associations between religiosity and different ER strategies did not vary by sample, except for expressive suppression. Religiosity was positively associated with expressive suppression in the Turkish sample but not in the American or Israeli samples. In this study, religiosity was associated with more cognitive reappraisal and more positive reframing but not with expressive suppression or venting in the Israeli sample.

Finally, using a sample of 1059 university students divided into different religions, it was found that atheists were more likely to suppress their emotions than religiously affiliated individuals; for cognitive reappraisal, no differences were found (Burris, 2022).

#### Associations Between Religion and ER

In a cross-sectional study with 210 inpatients waiting for surgery, intrapersonal religious commitment (with cognitive focus) was negatively associated with cognitive reappraisal (*r* = −0.40, *p* < 0.01) and positively associated with expressive suppression (*r* = 0.43, *p* < 0.001); interpersonal religious commitment (with a behavioral focus) was positively associated with cognitive reappraisal (*r* = 0.50, *p* < 0.001) and negatively associated with expressive suppression (*r* = −0.72, *p* < 0.01) (Aliche et al., 2020). Religious coping was positively associated with cognitive reappraisal (*r* = 0.25, *p* < 0.01) and negatively associated with expressive suppression (*r* = −0.14, *p* < 0.05) in a sample of 203 young adults (Dolcos et al., 2021).

In one longitudinal study with 1132 university students, higher involvement in religious activities was associated with fewer difficulties in ER over time (*β* = −0.06, *p* < 0.05). Spiritual attitudes/beliefs towards the sacred were not associated with ER (Semplonius et al., 2015). Also, in a longitudinal study with 167 adolescents (most of them protestants), religiousness was positively correlated with ER (as measured by the Emotion Regulation Checklist) (except for the organizational dimension of religiousness (*r* = 0.22, *p* < 0.01) (Holmes et al., 2019).

Intrinsic religiosity was positively associated with rumination (*r* = 0.18, *p* < 0.01) but not with ER in a sample of 326 adults, most of them Protestants and Catholics (Park & Yoo, 2016). In a sample of 150 students, high religious students use more functional cognitive ER strategies in comparison to low or medium religious students (Singh, 2014). In a sample of 220 adolescents (most of them Protestants), identification and introjection (two types of religious internalization) were associated (positively and negatively, respectively) with ER (as measured by the Emotion Regulation Checklist) (*b* = 0.09, *p* < 0.01 for identification and *b* = −0.11, *p* < 0.001 for introjection) (Holmes & Kim-Spoon, 2016).

In a study with 100 bereaved college students, it was found that religiousness and spirituality correlated with emotion reactivity (*r* = −0.30, *p* < 0.01; *r* = −0.24, *p* < 0.05, respectively) but not with emotion recovery (Lee et al., 2021).

#### ER as a Mediator

In one study, cognitive reappraisal mediated the link between religious coping and depression and anxiety (expressive suppression was not a significant mediator) (Dolcos et al., 2021). In a longitudinal study with university students, higher involvement in religious activities was associated with fewer difficulties in ER which in turn were associated with more social ties over time (Semplonius et al., 2015). Cognitive reappraisal (and expressive suppression only in a Christian sample) mediated the link between religiosity and life satisfaction (Vishkin et al., 2019a, 2019b). In one longitudinal study with adolescents, ER (at time 2) mediated the link between religiousness (at time 1) and risk-taking behavior (at time 3) (Holmes et al., 2019).

Emotional intelligence (in terms of appraisal of self-emotions and regulation and use of emotions) was a significant mediator in the association between religiousness and cyberbullying in university students; appraisal of others’ emotions was not a significant mediator (Yadav & Yadav, 2018). Rumination (but not ER) mediated the link between religiosity and the search for meaning and the presence of meaning (Park & Yoo, 2016).

ER was a significant mediator in the association between spiritual well-being and quality of life, mental health, and burnout (Akbari & Hossaini, 2018). Finally, self-regulation (that included ER but also behavioral and cognitive regulation) mediated the link between identification and introjection and health-risk behavior (Holmes & Kim-Spoon, 2016).

## Discussion

The main aim of this review was to examine findings from quantitative studies focused on exploring the link between religion/spirituality and ER. Overall, the results showed that this link has been explored especially from two points of view: one in terms of associations between religion/spirituality and ER according to religious affiliations, and the other in terms of associations between religion/spirituality and ER, regardless of religious affiliation. Most of the studies from this last view, also explored if ER potentially mediated the links between religion/spirituality and diverse psychological outcomes, which provided a processual approach to understanding these relationships.

It is important to note that despite the association between religion/spirituality and emotions having been recognized and theoretical proposed several years ago (e.g., Emmons, 2005), only recently studies started to test empirically these associations. Indeed, most of the included studies were conducted in the last few years (since 2014).

In terms of associations between religion/spirituality and ER according to religious affiliations, only five studies were found (Burris, 2022; Vishkin et al., 2016, 2019a, 2019b; Wilken & Miyamoto, 2020). These studies included data from four different religions (i.e., Buddhism, Islam, Christianity, or Judaism) and showed that there are indeed some small to moderate differences in ER according to religion. While religiosity was positively related to cognitive reappraisal in three different religious affiliations (Islam, Christianity, and Judaism), the same pattern was not found for expressive suppression since religiosity was not associated (for Muslims), was positively associated (for Jewish) or was negatively associated (for Christians) with expressive suppression.

It is important to note that the three religions under study are monotheistic religions, so no generalizations can be made for polytheistic religions. Also, no comparisons were made with agnostics or atheists which limits the conclusions of these studies. Only one study focused on atheists and suggested that these individuals tend to use more expressive suppression in comparison to religiously affiliated and agnostic/nonreligious individuals (Burris, 2022) but no differences were found in terms of cognitive reappraisal. According to Burris (2022), the difference in expressive suppression would be linked to a diminished acceptance of religious/spiritual experiences.

For the other studies that did not consider religious affiliation, overall, religiosity seemed to shape ER (but this was not the main objective for most of the included studies because they aimed to test the potential mediating role of ER).

There are several issues that should be considered to better understand the results obtained. First, it is important to note that the operationalization of religion/spirituality was very different among included studies. These included intrapersonal/intrinsic or interpersonal/extrinsic religiosity, religious coping, involvement in religious activities, religiousness, and even religiosity intensity. However, regardless of the measure used, studies showed that religion/spirituality influenced ER strategies, is usually linked to the use of more adaptative ER (such as cognitive reappraisal), negatively linked to the use of more desadaptive ER (such as expressive suppression), and associated with fewer difficulties in ER (e.g., Aliche et al., 2020; Dolcos et al., 2021; Semplonius et al., 2015; Singh, 2014), better ER (in terms of socially appropriate emotional displays, empathy, and emotional self-awareness) (Holmes et al., 2019), and higher emotional intelligence (Yadav & Yadav, 2018).

It seems that religion/spirituality facilitates cognitive reappraisal since it fosters finding meaning, control, and resilience (Dolcos et al., 2021). As pointed out by some authors, meaning making is one important facet of religions to help individuals to deal with fundamental questions and shape their thoughts and behaviors (e.g., Davies, 2011). Thus, promoting cognitive reappraisal contributes to changing individuals’ emotional experiences (Vishkin et al., 2016). Also, religious factors such as the community support or meaning framework related to religious beliefs seem to contribute to facilitating ER (Singh, 2014).

Thus, individuals with higher religiosity, regardless of religious affiliation, tend to regulate better their emotions (Vishkin et al., 2019a, 2019b). Additionally, for younger individuals, it seems that religion/spirituality is an important factor for promoting ER development (Holmes et al., 2019). Also, being involved in religious activities seems to contribute to improving ER which can be associated with the fact that faith-based activities can facilitate ER experiences in comparison to other community activities promoting intra and interpersonal regulation (Larson et al., 2006; Semplonius et al., 2015).

In one study, however, intrinsic religiosity was not linked to ER (Park & Yoo, 2016). The authors believe that the benefits associated with religiosity, including those related to ER, tend to increase with age and may not be presented in their young sample. Also, as pointed out by the authors, they assessed ER with a questionnaire focused on ER difficulties which can limit their conclusions (Park & Yoo, 2016). In this same study, intrinsic religiosity was associated with deliberative rumination, suggesting that intrinsic religiosity may be particularly useful under adverse conditions.

When ER was explored as a mediator, most of the studies confirmed that religion/spirituality promotes positive outcomes through facilitating ER. Overall, religion/spirituality contributes to improving individuals’ abilities to regulate their emotions which in turn facilitates psychological adaptation, namely in terms of protecting risk taking behaviors in adolescents (Holmes et al., 2019), promoting meaning finding, life satisfaction, quality of life, and psychological health (Akbari & Hossaini, 2018; Park & Yoo, 2016; Vishkin et al., 2019a, 2019b), preventing anxiety, depression, and burnout (Akbari & Hossaini, 2018; Dolcos et al., 2021). Thus, results suggest that ER is one important linking mechanism between religion/spirituality and psychological outcomes.

### Study Limitations

It is important to highlight two types of limitations. First, those related to the included studies and those related to the systematic review itself. In terms of the included studies, it is important to note few studies were found (suggesting that this topic is understudied), that most of them were cross-sectional in nature (which limits conclusions regarding causality among religion/spirituality and ER), and were conducted with very different participants (e.g., adolescents, young adults, adults, mourners), in different contexts and using different self-reported measures of religion and spirituality. Thus, findings and conclusions should be interpreted with caution.

Also, most of the studies focused on two specific ER strategies (i.e., cognitive reappraisal and expressive suppression), thus it is difficult to know what happens with other ER strategies.

In terms of limitations of the systematic review, it is important to highlight that it does not include grey literature which can lead to some reporting bias.

### Future Research

The overview of these results allows identifying some potential avenues for future research. First, differences in ER should be examined in other religions since we only found studies comparing four different religions (i.e., Buddhism, Islam, Christianity, or Judaism). Second, studies should explore other ER strategies besides cognitive reappraisal and expressive suppression (there is some evidence that religion/spirituality can also influence other strategies like rumination). Third, more longitudinal studies should be conducted to provide an overview of causality among religion/spirituality and ER.

Additionally, studies should try to develop a more consensual measure of religion/spirituality since most of the studies used different measures and strategies to assess religion/spirituality. Indeed, several studies had to adapt measures that can influence the results obtained.

## Conclusion

This review explored the link between religion/spirituality and ER in quantitative studies. Overall, small to moderate correlations found indicate that religion/spirituality seems positively influence ER by promoting adaptive strategies like cognitive reappraisal and reducing maladaptive strategies such as expressive suppression. These effects seem consistent across various operationalizations of religion/spirituality, including intrinsic religiosity, religious coping, and involvement in religious activities. Importantly, ER frequently mediated the relationship between religion/spirituality and positive psychological outcomes, such as increased resilience, reduced anxiety and depression, and improved life satisfaction.

In sum, religion/spirituality appears to play an important role in shaping ER and its impact on well-being. Future research should address this field’s diversity and investigate these processes across different populations and cultural contexts to deepen our understanding on these processes.
